# Discovery of Primarolides A and B from Marine Fungus *Asteromyces cruciatus* Using Osmotic Stress and Treatment with Suberoylanilide Hydroxamic Acid

**DOI:** 10.3390/md17080435

**Published:** 2019-07-24

**Authors:** Hope A. Igboeli, Douglas H. Marchbank, Hebelin Correa, David Overy, Russell G. Kerr

**Affiliations:** 1 Department of Chemistry, University of Prince Edward Island, Charlottetown, PE C1A 4P3, Canada; 2 Nautilus Biosciences Croda, Regis and Joan Duffy Research Centre, 550 University Avenue, Charlottetown, PE C1A 4P3, Canada; 3 Department of Pathology and Microbiology, Atlantic Veterinary College, University of Prince Edward Island, Charlottetown, PE C1A 4P3, Canada; 4 Department of Biomedical Science, Atlantic Veterinary College, University of Prince Edward Island, Charlottetown, PE C1A 4P3, Canada

**Keywords:** natural product, epigenetic modification, silent biosynthetic gene clusters, metabolomics, *Asteromyces cruciatus*

## Abstract

Advances in whole-genome sequencing of many fungal species has revealed the presence of numerous “silent” biosynthetic genes, highlighting their potential to produce a wide variety of natural products. These silent biosynthetic genes are regulated in part by their highly condensed chromatin structure, which can be modified to allow transcription in response to external stimuli. In this study, *Asteromyces cruciatus* was subjected to both epigenetic modification and osmotic stress to enhance the production of new natural products. This “cooperative induction” strategy led to the isolation and characterization of two new polyketides from a fermentation of *A. cruciatus* treated with suberoylanilide hydroxamic acid and sodium chloride. The metabolic profiles of the control and treated samples were assessed using ultra-high performance liquid chromatography high-resolution electrospray ionization mass spectrometry (UHPLC-HRESIMS) metabolomic analysis, highlighting the upregulation of two new polyketides, primarolides A and B. These compounds were purified using reversed-phase flash chromatography followed by high-performance liquid chromatography, and their planar structures were established using NMR spectroscopy.

## 1. Introduction

Filamentous fungi are well-known for their ability to produce a vast array of natural products that exhibit a range of biological activities, and many of these compounds, including penicillin, cyclosporine, and lovastatin, have had a major impact in human medicine [1,2]. While natural products continue to be an excellent source of compounds with potential therapeutic applications, the rate of new natural product discovery has slowed. Meanwhile, advances in genome sequencing and gene annotation have revealed that many fungi have the genetic potential to produce more natural products than what is commonly observed under standard fermentation conditions [2,3,4,5]. Inducing the expression of these “silent” natural product biosynthetic gene clusters could therefore provide access to a vast untapped reservoir of new natural products [3,4,6]. Working toward this aim, several strategies have been investigated, including, for instance, the overexpression of regulatory genes such as *laeA*. This gene encodes for a methyltransferase that serves as a global regulator of secondary metabolism in several fungi, and its activation has successfully led to the discovery of new natural products [7,8]. Overexpression of pathway-specific activator genes has also been a successful approach and has led to the isolation of previously unknown aspyridone from *Aspergillus nidulans* [4].

Co-cultivation is known to induce the expression of silent natural products, as demonstrated recently by Nützmann et al. in a co-culture of *A. nidulans* and a soil-dwelling bacterium *Streptomyces rapamycinicus* [2]. This co-culture led to activation of the orsellinic acid gene cluster in *A. nidulans* and increased production of orsellinic acid and the structurally related metabolite lecanoric acid [2]. The mechanism of *ors* activation was shown to involve histone modification through an increase in histone 3 acetylation via the Saga/Ada complex. Histone acetylation weakens the electrostatic interaction between histone proteins and negatively charged DNA, resulting in chromatin that is more accessible for transcription. The involvement of histone acetylation in *orsA* expression was further corroborated through the addition of histone acetyltransferase (HAT) inhibitor anacardic acid and histone deacetyltransferase (HDAC) inhibitor suberoylanilide hydroxamic acid (SAHA), which blocked and activated *orsA* expression, respectively [2]. These studies point toward the potential utility of SAHA and other small molecules as epigenetic modifiers to modulate histone-regulating enzymes in order to activate silent natural product biosynthetic gene clusters in a relatively high-throughput manner [3,9,10].

Another approach to activating the expression of silent natural products in fungi is with the use of abiotic stress [11,12]. Our group previously showed in *Aspergillus aculeatus* that osmotic and saline stress exerted by glycerol and NaCl, respectively, can modulate secondary metabolism, causing some metabolites to increase or decrease relative to the concentration of the osmolite [11]. Osmotic stress is known to trigger the high osmolarity glycerol (HOG) mitogen-activated protein kinase (MAPK) pathway, which in turn induces the transcription of genes involved in the adaptive response to osmotic stress. Such genes include glycerol-3-phosphate dehydrogenase *Gpd1p* that encodes for an enzyme involved in glycerol biosynthesis, which helps maintain osmotic balance [13]. However, changes in gene expression in response to osmotic stress may also lead to changes in the expression of natural product biosynthetic gene clusters.

We report herein the use of epigenetic modifiers, SAHA and 5-azacytidine, and abiotic stress, including osmotic and oxidative stress, to induce or upregulate the biosynthesis of natural products in *Asteromyces cruciatus* (family Dermatiaceae, order Helotiales). We also examine the potential impact of combining epigenetic modification and abiotic stress on natural product production. Fermentation extracts were subjected to ultra-high performance liquid chromatography high-resolution electrospray ionization mass spectrometry (UHPLC-HRMS) analysis and the data was processed using a metabolomics approach to identify changes in secondary metabolite production between the different treatments. We report herein that the combined use of SAHA and osmotic stress resulted in a “cooperative” upregulation of secondary metabolites, leading to the identification of new polyketides, primarolides A (**1**) and B (**2**).

## 2. Results and Discussion

### 2.1. Treatment with SAHA and NaCl Significantly Upregulated Primarolides A and B

**Figure 1:**
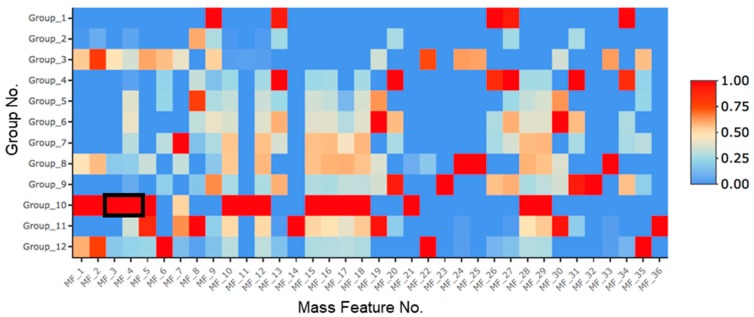
Heat map of UHPLC-HRESIMS metabolomics data showing mass features (MF) significantly different than the control groups (*n* = 3). The rows represent each of the treatment conditions as defined in Table 1, while the horizontal axis represents significant mass features (*m/z*_R_t_) summarized in Table S1. Peak areas were normalized with blue cells representing peak areas less than or equal to the detection threshold (2 × 10^4^), while red cells represent peak areas in the treatment with the highest production level. The black box highlights mass features representing primarolides A (**1**) and B (**2**).

*Asteromyces cruciatus* was fermented in 12 treatment conditions, as shown in Table 1, to explore the metabolic response of *A. cruciatus* to epigenetic modification, abiotic stress, and both in combination. Extracts derived from these cultures were analyzed using ultra-high performance liquid chromatography high-resolution electrospray ionization mass spectrometry (UHPLC-HRESIMS) to identify metabolites whose production was induced or upregulated in response to these treatments. In total, 1189 mass features were identified. Statistical analysis of the data matrix led to the identification of 38 mass features that were significantly different from the control treatments. A heat map showing the presence of these mass features across the different treatment conditions was created (Figure 1). In these fermentations, we observed two mass features 369.1333_3.33 (*m*/*z*_R_t_) and 444.1802_3.49, representing unknown compounds later determined to be primarolides A (**1**) and B (**2**). The highest production of these metabolites was observed in fermentations containing a combination of NaCl and SAHA, where both compounds were significantly upregulated. Interestingly, a low production of **1** was observed in treatments containing NaCl and SAHA on their own. Meanwhile, **2** was not detected at all in the control and NaCl treatments.

**Figure 2:**
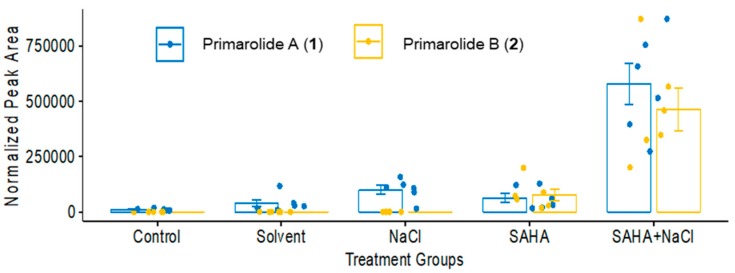
Production of primarolides A (**1**) and B (**2**) across five treatment conditions (*n* = 6) represented by normalized peak areas.

The fermentations were repeated to confirm the cooperative effect of SAHA and NaCl on the production of **1** and **2**. This experiment focused on treatment conditions in groups 1, 3, 7, and 10 (Table 1). Mass features belonging to **1** and **2** (Figure 2) were evaluated using one-way ANOVA (F value = 29, Tables S2 and S3), and then Tukey’s honestly significant difference (HSD) test (Tables S4 and S5) was used to determine which treatment pairs were significantly different from each other. With respect to both **1** and **2**, treatments containing SAHA and NaCl alone were not significantly different from the control. However, **1** and **2** were both significantly higher in the SAHA + NaCl treatment, confirming the previously observed “cooperative” upregulation. A database search of these mass features on AntiBase 2017 (Wiley) indicated their potential novelty as there were no known natural products with predicted *m*/*z* values within 5 ppm of these observed mass peaks. These compounds were therefore investigated further to determine their chemical structures.

### 2.2. Purification and Characterization of Primarolides A and B

**Figure 3:**
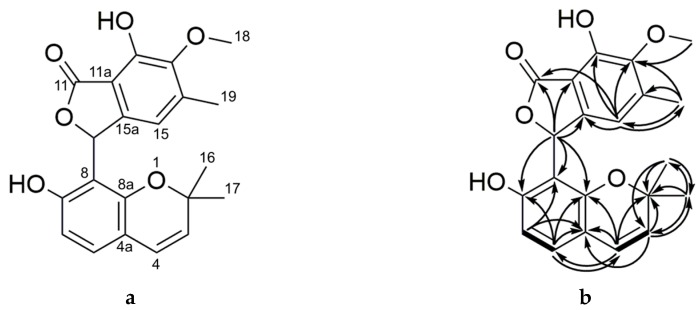
Chemical structure of primarolide A (**1**) showing (**a**) numbered positions and (**b**) key HMBC (^1^H → ^13^C) and COSY (bold bonds) correlations.

Compound **1** (primarolide A) was purified from the crude extract of *A. cruciatus* using semi-preparative RP-HPLC. The molecular formula C_21_H_20_O_6_ was assigned on the basis of HRMS (*m*/*z* 369.1333 [M + H]^+^), indicating 12 degrees of unsaturation. The FTIR spectrum suggested the presence of carbonyl (1729 cm^−1^), aromatic C=C (1607 cm^−1^), and hydroxyl (3258 cm^−1^) functionalities. The ^1^H NMR spectrum showed signals for two methyl singlets H-16 (δ_H_ 0.65) and H-17 (δ_H_ 1.17), which exhibited HMBC correlations with C-17 (δ_C_ 27.9) and C-16 (δ_C_ 26.8), respectively, indicating the presence of a geminal dimethyl group (Table 2). An HMBC correlation between H-16/H-17 and C-2 (δ_C_ 77.2) established the attachment of these two methyl groups to an oxygenated quaternary carbon. A COSY correlation between olefinic protons H-3 [δ_H_ 5.33 (1H, d, *J* = 9.8 Hz)] and H-4 [δ_H_ 6.16 (1H, d, *J* = 9.8 Hz)] showed the presence of a double bond. Additional HMBC correlations between H-16/H-17 and C-3, and between H-3 and C-2/C-17, confirmed the position of the double bond relative to the geminal dimethyl group. Quaternary carbon resonances C-4a (δ_C_ 115.4) and C-8a (δ_C_ 154.7) were assigned using their HMBC correlations with H-3 and H-4. The downfield chemical shift of the latter carbon resonance indicated it was oxygenated, suggesting the presence of a pyran ring (Figure 3).

A COSY correlation was observed between aryl protons H-5 [δ_H_ 6.42 (1H, d, *J* = 8.3 Hz)] and H-6 [δ_H_ 6.42 (1H, d, *J* = 8.3 Hz)]. An HMBC correlation between H-4 and C-5 (δ_C_ 128.7) confirmed the attachment of C-4 to C-5. Meanwhile, H-5 showed an HMBC correlation with C-7 (δ_C_ 157.8), revealing the presence of an aromatic hydroxyl group adjacent to H-6. Additional HMBC correlations between H-6/C-8 and H-5/C-8a suggested the presence of a 2,2-dimethyl-1-benzopyran partial structure. A methine proton at H-9 [δ_H_ 6.99 (1H, br s)] also showed HMBC correlations with C-7, C-8, and C-8a to further corroborate this assignment. Given the downfield chemical shifts of H-9 and C-9 (δ_C_ 74.9), this position must be oxygenated. Further HMBC correlations between H-9 and C-11a (δ_C_ 144.2) and C-15a (δ_C_ 113.6) indicated the presence of another aromatic ring. Further analysis of HMBC correlations involving aryl proton H-12 (δ_H_ 6.72), methyl H-18 (δ_H_ 2.23), and methoxy H-19 (δ_H_ 3.43) confirmed the substitution pattern of this aromatic ring. The position of carbonyl C-11 (δ_C_ 173.0) was confirmed using HMBC correlations with H-9. The remaining degree of unsaturation was attributed to the presence of a γ-lactone moiety, thus completing the planar structure of primarolide A (**1**).

**Figure 4:**
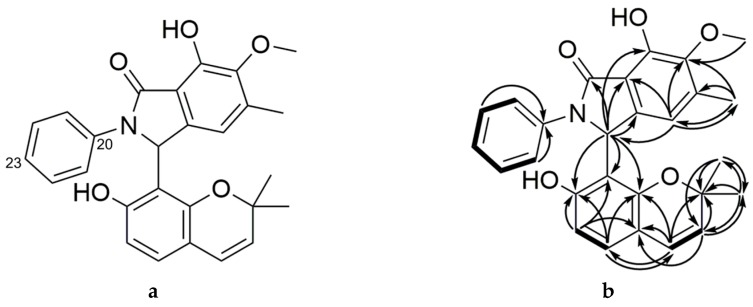
Chemical structure of Primarolide B (**2**) showing (**a**) numbered positions and (**b**) key HMBC (^1^H → ^13^C) and COSY (bold bonds) correlations.

Primarolide B (**2**) was purified alongside **1** using semi-preparative RP-HPLC. The molecular formula of **2** was assigned as C_27_H_25_O_5_N on the basis of HRESIMS (*m*/*z* 444.1802 [M + H]^+^), indicating 16 degrees of unsaturation. NMR spectroscopic data for primarolide B (Table 3) was similar to **1**, except the γ-lactone was replaced with a *N*-phenyl γ-lactam (Figure 4). Notably, the upfield chemical shift of C-9 (δ_C_ 56.1) was consistent with this assignment. The *N*-phenyl moiety of **2** is uncommon in natural products and may be derived from the aniline moiety of SAHA. Experiments are underway to confirm the involvement of SAHA in the formation of **2** and these results will be described elsewhere. Attempts to form crystals to determine the absolute configurations of **1** and **2** at position C-9 using X-ray crystallography were unsuccessful. Massarinins A and B are among the most structurally related natural products to **1** and **2**, although they both lack the γ-lactone and -lactam rings found in **1** and **2** [14]. These metabolites were reported to have activity against *Staphyloccocus aureus* at 200 µg/disk in disk diffusion assays. However, primarolides A and B showed no significant activity in microplate antimicrobial assays against methicillin-resistant *S. aureus* (MRSA), *Staphylococcus warneri*, vancomycin-resistant *Enterococcus faecium* (VRE), *Pseudomonas aeruginosa*, *Proteus vulgaris*, and *Candida albicans* at the highest test concentration of 128 µg/mL. This result suggests that the 2,2-dimethyl-1-benzopyran moiety did not mediate the antimicrobial activity of the massarinins.

A key challenge in modern natural product research is to develop an understanding of factors that induce the expression of natural product biosynthetic gene clusters that are otherwise silent under standard laboratory culture conditions. Epigenetic modifiers have recently been utilized to induce differential gene expression in fungi, resulting in the identification of many new natural products. For example, eupenicinicols C and D were isolated from an endophytic fungus *Eupenicillium* sp. in fermentations containing the epigenetic modifier nicotinamide, which is a NAD^+^-dependent HDAC inhibitor [15]. Previous studies have also shown that the concerted action of HDAC and DNA methyltransferase inhibitors can induce secondary metabolism. The addition of SAHA and 5-azacytidine to the culture medium of *Isaria tenuipes* led to the isolation of tenuipyrone, which was not observed in fermentations treated with SAHA or 5-azacytidine alone [16]. This result highlights that the concerted action of these two epigenetic modifiers was required to induce the expression of the tenuipyrone biosynthesis.

Rodrigues et al. have demonstrated that osmotic stress brought on by higher concentrations of KCl in the culture medium can induce differential gene expression, including the activation of HOG1 and enzymes involved in glycerol production [13]. Our recent investigation into the effects of glycerol and NaCl on *A. aculeatus* showed that osmotic stress can also modulate the production of natural products in fungi of either marine or terrestrial origin [11]. We demonstrate herein that the combination of SAHA and NaCl can lead to the upregulation of secondary metabolites, facilitating the isolation and identification of new natural products. Interestingly, primarolide A (**1**) was not produced in significant quantities when *A. cruciatus* was treated with SAHA or NaCl alone. These findings suggest that the expression of natural products can be significantly increased by the cooperative effects of epigenetic modifiers and osmotic stress.

## 3. Materials and Methods

### 3.1. General Experimental Procedures

Infrared (IR) spectra were recorded using attenuated total reflectance on a Thermo Nicolet 6700 FTIR spectrometer (Thermo Scientific, Waltham, MA, USA). All ^1^H, ^13^C, and two-dimensional NMR spectra were acquired on a 600 MHz Bruker Avance III NMR spectrometer (Bruker, Billerica, MA, USA) equipped with a 5 mm cryoprobe. All chemical shifts are reported in ppm and referenced to the residual solvent signal of CD_3_OD (^1^H: 3.31 ppm; ^13^C: 49.1 ppm). Coupling constants are reported in Hz with the following abbreviations: singlet (s), doublet (d), triplet (t), doublet of doublets (dd), broad (br), apparent (app.). UHPLC-HRMS analyses were carried out using a Thermo Accela chromatograph (Thermo Scientific, Waltham, MA, USA) equipped with HRMS-ELSD-UV detection, which included a Thermo LTQ Exactive (Thermo Scientific, Waltham, MA, USA) fitted with an ESI source, Sedex 80 LT-ELSD (Sedere, Olivet, France), and Thermo PDA (Thermo Scientific, Waltham, MA, USA). For all UHPLC-HRMS analysis, the following chromatographic conditions were used: column, Kinetex Core-Shell 100 Å (2.1 × 50 mm, 1.7 μm, Phenomenex, Torrance, CA, USA); mobile phase flow rate, 0.5 mL/min; injection volume, 10 μL; linear gradient, H_2_O:CH_3_CN (95:5, 0.1% formic acid) at 0.2 min to 100% CH_3_CN (0.1% formic acid) at 4.8 min, which was held until 8.0 min before returning to H_2_O:CH_3_CN (95:5, 0.1% formic acid) for 1.5 min. The following HRMS parameters were used: positive ionization mode; mass resolution, 30,000; mass range, *m/z* 190 to 2000; spray voltage, 2.0 kV; capillary temperature, 300 °C; S-lens RF voltage, of 60.0%; maximum injection time, 10 ms; 1 microscan. The system was controlled using Thermo Xcalibur 2.2 software modules (Thermo Scientific, Waltham, MA, USA). All solvents and reagents were purchased from commercial sources and used without further purification. All solvents used for purification were HPLC grade or higher.

### 3.2. A. cruciatus Isolation, Fermentation, and Extraction

The fungus *A. cruciatus* was isolated from a sea foam sample collected on the coast of Point Prim, Prince Edward Island, Canada. Cryogenic vials containing fungal mycelia in 12.5% (*v*/*v*) glycerol stored at −80 °C were thawed, inoculated into 15 mL SMYA liquid medium at 22 °C and agitated at 200 rpm for 7 days to prepare the seed cultures. Seed cultures (500 μL) were then inoculated in MMK2 medium for 14 days. For treatments containing H_2_O_2_ and NaCl, these reagents were added to achieve a final concentration of 100 nM and 1.25 M, respectively, before adding the inoculum. Epigenetic modifiers, SAHA and 5-azacytidine, were added after 4 days at two different concentrations (10 and 100 µM) using DMSO (100 µL), while all other treatments received a DMSO vehicle control (100 µL). The mycelia were macerated using 5 mm glass beads and extracted using ethyl acetate. Crude extracts were solubilized in CH_3_OH at a concentration of 500 μg/mL and analyzed using UHPLC-HRMS.

### 3.3. Metabolomics and Statistical Analysis

The metabolomics analysis was carried out using MZmine 2.37 (Okinawa Institute of Science and Technology, Okinawa, Japan) following a procedure previously described by our group [17]. Mass detection was completed with the full width at half maximum (FWHM) algorithm using a noise level threshold of 2 × 10^4^. Mass features associated with controls, which included fermentation media, media + SAHA, media + 5-azacytidine, and solvent blanks, were removed from the data matrix. The processed data matrix was loaded into R 0.99.491 using RStudio 0.99.491. Univariate and multivariate statistical analysis was carried out using the Metabolomic Univariate and Multivariate Analysis (MUMA) software package in R [18]. This analysis applied Shapiro Wilk’s test for normality (*p*-value > 0.05 for a normal distribution) to each variable and used either Welch’s *t*-test (parametric) or the Wilcoxon–Mann–Whitney test (non-parametric) to calculate *p*-values for each pairwise comparison.

### 3.4. Extraction and Purification of Primarolides A and B

A scale-up fermentation of *A. cruciatus* was completed by inoculating 15 mL culture tubes containing MMK2 medium. After growing for 14 days at 22 °C with shaking at 200 rpm, seed cultures were added to Fernbach culture flasks containing a total of 7 L MMK2 media. Cultures were grown for a total of 7 days at 22 °C with shaking at 200 rpm. SAHA and NaCl were added after 4 days to achieve a final concentration of 100 µM of SAHA and 1.25 M NaCl. The mycelia and broth were then separated and extracted twice using an equal volume of ethyl acetate. The extracts were combined and evaporated to dryness, yielding a 2.5 g crude extract. Fatty acids were removed by partitioning the crude extract between 80% CH_3_CN_(aq)_ and hexanes. The CH_3_CN layer was collected and evaporated to dryness to provide a 1.0 g crude extract, which was then fractionated using automated medium-pressure liquid chromatography with a CombiFlash Rf-200 chromatography system (Teledyne Isco, Lincoln, NE, USA) in a 50 g C_18_ column (RediSep Rf Gold^®^, Teledyne Isco, Lincoln, NE, USA). The mobile phase had a flow rate of 30 mL/min and a linear gradient from 5% CH_3_CN_(aq)_ to 100% CH_3_CN over 25 min. Fractions eluted at 15 min were combined, evaporated, and subjected to RP-HPLC on an Accella chromatography system (Thermo Scientific, Waltham, MA, USA). The method employed a semi-preparative Luna phenyl-hexyl column (10 × 250 mm, 5 μm, Phenomenex, Torrance, CA, USA) with an isocratic mobile phase consisting of 40% CH_3_CN_(aq)_ at a flow rate of 2.5 mL/min. This separation led to the purification of primarolides A (**1**, 33 min, 5 mg) and B (**2**, 40 min, 2 mg). All one- and two-dimensional NMR spectra are available in Figures S1–S13.

### 3.5. Antimicrobial Testing of Primarolides A and B

Primarolides A and B were tested for antimicrobial activity against methicillin-resistant *Staphylococcus aureus* (MRSA, ATCC 33591), *S. warneri* (ATCC 17917), vancomycin-resistant *Enterococcus faecium* (VRE, EF379), *Pseudomonas aeruginosa* (ATCC 14210), *Proteus vulgaris* (ATCC 12454), and *Candida albicans* (ATCC 14035). All testing was carried out in triplicate according to the Clinical Laboratory Standards Institute testing standards in a 96-well plate microbroth dilution assay, as previously described [19].

## 4. Conclusions

Two new polyketides, primarolides A (**1**) and B (**2**), were isolated and characterized from *A. cruciatus* fermentations treated with SAHA and high concentrations of NaCl. Treatment with SAHA or NaCl alone did not result in significant production of **1** and **2**. This study suggests that concomitant inhibition of HDAC and changes in gene expression induced by osmotic stress can amplify the expression of natural product biosynthetic gene clusters, which can aid the discovery of new natural products that may otherwise go undetected. While numerous studies have reported the isolation of new fungal secondary metabolites using epigenetic modifiers, to our knowledge, this study represents the first instance of combining epigenetic modification with osmotic stress to upregulate natural product biosynthesis.

As is evident from the heat map in Figure 1, in addition to the mass features corresponding to primarolides A and B, there are a number of other mass features that were induced/upregulated by treatment with SAHA and NaCl. This cooperative approach of combining two treatments is clearly the most effective approach of those used in this study to upregulate the production of natural products. Interestingly, there are also other mass features whose expression was affected by other single and cooperative treatments. Current efforts are underway to identify selected metabolites.

## Figures and Tables

**Table 1:** *A. cruciatus* fermentation treatment conditions.

Group No.	SAHA (µM)	5-aza (µM)	H_2_O_2_ (nM)	NaCl (M)
1	-	-	-	-
2	10	-	-	-
3	100	-	-	-
4	-	10	-	-
5	-	100	-	-
6	-	-	100	-
7	-	-	-	1.25
8	100	-	100	-
9	-	100	100	-
10	100	-	-	1.25
11	-	100	-	1.25
12	100	100	-	-

SAHA: suberoylanilide hydroxamic acid; 5-aza: 5-azacytidine.

**Table 2:** NMR spectroscopic data (^1^H NMR: 600 MHz, ^13^C NMR: 151 MHz, CD_3_OD) for primarolide A (δ in ppm relative to the residual solvent signal). ^*a*^

Position	δ_C_, Type		δ_H_, Mult (*J* in Hz)	COSY ^*b*^	HMBC ^*b*^
1					
2	77.2	C			
3	128.3	CH	5.33, d (9.8)	4	2, 4a, 17
4	122.7	CH	6.16, d (9.8)	3	2, 3, 4a, 5, 8a
4a	115.4	C			
5	128.7	CH	6.83, d (8.3)	6	4, 7, 8a,
6	108.4	CH	6.42, d (8.3)	5	4a, 8
7	157.9	C			
8	111.2	C			
8a	154.7	C			
9	75.0	CH	6.99, br s		7, 8, 8a, 11, 11a, 15a
10					
11	173.2	C			
11a	144.3	C			
12	153.4	C			
13	146.7	C			
14	141.6	C			
15	118.8	CH	6.72, br s		11, 12, 13, 15a
15a	113.6	C			
16	26.8	CH_3_	0.65, s		2, 3, 17
17	27.9	CH_3_	1.17, s		2, 3, 16
18	60.6	CH_3_	3.43, s		13
19	16.4	CH_3_	2.23, s		13, 14, 15

^*a*^ See Figures S1–S5 for NMR spectra. ^*b*^ COSY and HMBC correlations shown were from proton(s) stated with the indicated proton and carbon, respectively.

**Table 3:** NMR spectroscopic data (^1^H NMR: 600 MHz, ^13^C NMR: 151 MHz, CD_3_OD) for primarolide B (δ in ppm relative to the residual solvent signal). ^*a*^

Position	δ_C_, Type		δ_H_, Mult (*J* in Hz)	COSY ^*b*^	HMBC ^*b*^
1					
2	76.8	C			
3	127.8	CH	5.24, d, (9.7)	4	2, 4a, 16
4	122.9	CH	6.04, d, (9.7)	3	2, 4a, 5, 8a
4a	115.4	C			
5	127.5	CH	6.62, d, (8.2)	6	7, 8, 8a
6	108.4	CH	6.26, d, (8.2)	5	4a, 7, 8
7	158.4	C			
8	111.2	C			
8a	154.0	C			
9	56.1	CH	6.68, d (0.6)		7, 8, 8a, 11, 11a, 12, 15a
10					
11	168.9	C			
11a	132.1	C			
12	149.4	C			
13	149.9	C			
14	133.1	C			
15	122.2	CH	6.77, d (0.6)		9, 11a, 13, 14, 19
15a	126.9	C			
16	26.5	CH_3_	0.61, s		2, 3, 17
17	28.9	CH_3_	1.18, s		2, 3, 16
18	15.6	CH_3_	2.28, s		13, 14, 15
19	62.5	CH_3_	3.96, s		13
20	139.3	C			
21	124.1	CH	7.69, app. d (8.5)	22	20, 22
22	129.3	CH	7.27, app. dd (7.5, 8.5)	21, 23	20, 23
23	125.9	CH	7.07, app. t (7.5)	22	21, 22

^*a*^ See Figures S7–S12 for NMR spectra. ^*b*^ COSY and HMBC correlations shown were from proton(s) stated with the indicated proton and carbon, respectively.

## Supplementary Materials

The following are available online at https://www.mdpi.com/1660-3397/17/8/435/s1, Table S1: Summary of mass features shown in the heat map in Figure 1; Table S2: One-way ANOVA test (primarolide A); Table S3: One-way ANOVA test (primarolide B); Table S4: Tukey’s HSD Test (primarolide A); Table S5: Tukey’s HSD test (primarolide B); Figure S1: ^1^H NMR spectrum (600 MHz, CD_3_OD) of primarolide A (**1**); Figure S2: ^13^C NMR spectrum (150 MHz, CD_3_OD) of primarolide A (**1**); Figure S3: COSY NMR spectrum of primarolide A (**1**); Figure S4: HSQC NMR spectrum of primarolide A (**1**); Figure S5: HMBC NMR spectrum of primarolide A (**1**); Figure S6: FTIR spectrum of primarolide A (**1**); Figure S7: ^1^H NMR spectrum (600 MHz, CD_3_OD) of primarolide B (**2**); Figure S8: ^13^C NMR spectrum (150 MHz, CD_3_OD) of primarolide B (**2**); Figure S9: COSY NMR spectrum of primarolide B (**2**); Figure S10: HSQC NMR spectrum of primarolide B (**2**); Figure S11: HMBC (8 Hz) NMR spectrum of primarolide B (**2**); Figure S12: HMBC (3 Hz) NMR spectrum of primarolide B (**2**); and Figure S13: FTIR spectrum of primarolide B (**2**).
