# Supplementary material for: Discovery of Primarolides A and B from Marine Fungus Asteromyces cruciatus Using Osmotic Stress and Treatment with Suberoylanilide Hydroxamic Acid
Source: Mar Drugs. 2019 Jul 24;17(8):435. doi: 10.3390/md17080435 (PMC6723326; doi:10.3390/md17080435)
Supplement: Supplementary file 1 [file marinedrugs-17-00435-s001.pdf]

Supplementary Material

Discovery of Primarolides A and B from Marine Fungus *Asteromyces cruciatus* by Osmotic Stress and Treatment with Suberoylanilide Hydroxamic Acid

Hope A. Igboeli [1,*], Douglas H. Marchbank [1,3,*], Hebelin Correa [3], David Overy [1,4] and Russell G. Kerr [1,2,3,†]

1 Department of Chemistry, University of Prince Edward Island, Charlottetown, PE, Canada, C1A 4P3.

2 Department of Biomedical Science, Atlantic Veterinary College, University of Prince Edward Island, Charlottetown, PE, Canada, C1A 4P3.

3 Nautilus Biosciences Croda, Regis and Joan Duffy Research Centre, 550 University Avenue, Charlottetown, PE, Canada, C1A 4P3.

4 Department of Pathology and Microbiology, Atlantic Veterinary College, University of Prince Edward Island, Charlottetown, PE, Canada, C1A 4P3.

* These authors contributed equally to this work.

† Correspondence: rkerr@upei.ca; Tel.: +1-902-566-0565

Table of Contents

Table S1. Summary of mass features shown in the heat map in **Figure 1**....................................3

Table S2. One-way ANOVA Test (Primarolide A)..4

Table S3. One way ANOVA test (Primarolide B)...4

Table S4. Tukey's HSD Test (Primarolide A) 4

Table S5. Tukey's HSD Test (Primarolide B)...5

Figure S1. ^{1}H NMR spectrum (600 MHz, CD_3OD) of Primarolide A (**1**) 6

Figure S2. ^{13}C NMR spectrum (150 MHz, CD_3OD) of Primarolide A (**1**)..7

Figure S3. COSY NMR spectrum of Primarolide A (**1**)...8

Figure S4. HSQC NMR spectrum of Primarolide A (**1**) 9

Figure S5. HMBC NMR spectrum of Primarolide A (**1**) 10

Figure S6. FTIR spectrum of Primarolide A (**1**) 11

Figure S7. ^{1}H NMR spectrum (600 MHz, CD_3OD) of Primarolide B (**2**).......................................12

Figure S8. ^{13}C NMR spectrum (150 MHz, CD_3OD) of Primarolide B (**2**) 13

Figure S9. COSY NMR spectrum of Primarolide B (**2**) 14

Figure S10. HSQC NMR spectrum of Primarolide B (**2**)..15

Figure S11. HMBC (8 Hz) NMR spectrum of Primarolide B (**2**)...16

Figure S12. HMBC (3 Hz) NMR spectrum of Primarolide B (**2**)...17

Figure S13. FTIR spectrum of Primarolide B (**2**) 18

Table S1. Summary of mass features shown in the heat map in **Figure 1**.

**Mass ID**	***m/z*_RT**
MF_1	200.11_3.48
MF_2	216.10_2.90
MF_3	444.18_3.50
MF_4	369.13_3.33
MF_5	277.22_4.51
MF_6	300.17_2.44
MF_7	317.21_4.49
MF_8	327.22_4.54
MF_9	353.23_3.25
MF_10	367.36_6.12
MF_11	375.23_3.32
MF_12	385.37_6.12
MF_13	388.28_4.01
MF_14	394.32_4.67
MF_15	395.39_6.93
MF_16	402.39_6.11
MF_17	413.38_5.86
MF_18	429.37_6.65
MF_19	549.13_2.65
MF_20	558.30_4.15
MF_21	569.31_3.51
MF_22	635.41_4.39
MF_23	659.39_3.77
MF_24	781.53_4.35
MF_25	803.58_4.33
MF_26	954.62_3.61
MF_27	388.28_4.01
MF_28	402.39_6.11
MF_29	429.37_6.66
MF_30	549.13_2.65
MF_31	558.30_4.15
MF_32	659.39_3.77
MF_33	781.53_4.35
MF_34	954.62_3.61
MF_35	300.17_2.44
MF_36	377.29_4.66

Abbreviations: MF: mass feature; *m*/*z*: mass to charge ratio; RT: retention time

Table S2. One-way ANOVA Test (Primarolide A).

	DF	SUM SQ	MEAN SQ	F-VALUE	Pr>F
Treatment	4	1.347e+12	3.367e+11	29.44	3.94e-09 ***
Residuals	25	2.859e+11	1.144e+10		

Significant codes: 0 '***' 0.001 '**' 0.01 '*' 0.05 '.' 0.1 ' ' 1. Abbreviations DF = degree of freedom, SUM SQ = sum of squares, Mean SQ = mean squares, F-VALUE = Fisher statistics value (F value = 1 indicates no variability between means), Pr > F = probability of the F statistics.

Table S3. One-way ANOVA test (Primarolide B).

	DF	SUM SQ	MEAN SQ	F-VALUE	Pr>F
Treatment	4	9.660e+11	2.415e+11	20.16	1.58e-07 ***
Residuals	25	2.995e+11	1.198e+10		

Significant codes: 0 '***' 0.001 '**' 0.01 '*' 0.05 '.' 0.1 ' ' 1. Abbreviations DF = degree of freedom, SUM SQ = sum of squares, Mean SQ = mean squares, F-VALUE = Fisher statistics value (F value = 1 no variability between means), Pr > F = probability of the F statistics.

Table S4. Tukey's HSD Test (Primarolide A).

Treatment	Difference	Lower limit	Upper limit	Adjusted P-value
Solvent control versus control	28953.97	-152381.56	210289.5	0.9894786
SAHA versus control	52078.87	-129256.65	233414.4	0.9143093
NaCl versus control	89961.41	-91374.12	271296.9	0.5983612
SAHA+ NaCl versus control	567321.92	385986.39	748657.4	0.0000000
SAHA Versus Solvent control	23124.90	-158210.62	204460.4	0.9955347
NaCl versus solvent control	61007.44	-120328.09	242343.0	0.8581530
SAHA+ NaCl versus Solvent control	538367.95	357032.42	719703.5	0.0000000
NaCl versus SAHA	37882.53	-143452.99	219218.1	0.9715838
SAHA+ NaCl versus SAHA	515243.04	333907.52	696578.6	0.0000001
SAHA+ NaCl versus NaCl	477360.51	296024.98	658696.0	0.0000004

This analysis was done using normalized peak area from treatments with SAHA, NaCl and SAHA + NaCl (n = 6). Adjusted p-value < 0.005 indicates treatments with significant variability between means.

Table S5. Tukey's HSD Test (Primarolide B).

Treatment	Difference	Lower limit	Upper limit	Adjusted P-value
Solvent control versus control	-5.335702e-11	-185599.2	185599.2	1.0000000
SAHA versus control	7.715992e+04	-108439.2	262759.1	0.7395279
NaCl versus control	1.358179e-10	-185599.2	185599.2	1.0000000
SAHA+ NaCl versus control	4.616350e+05	276035.8	647234.1	0.0000011
SAHA Versus Solvent control	7.715992e+04	-108439.2	262759.1	0.7395279
NaCl versus solvent control	1.891749e-10	-185599.2	185599.2	1.0000000
SAHA+ NaCl versus Solvent control	4.616350e+05	276035.8	647234.1	0.0000011
NaCl versus SAHA	-7.715992e+04	-262759.1	108439.2	0.7395279
SAHA+ NaCl versus SAHA	3.844750e+05	198875.9	570074.2	0.0000216
SAHA+ NaCl versus NaCl	4.616350e+05	276035.8	647234.1	0.0000011

This analysis was done using normalized peak area from treatments with SAHA, NaCl and SAHA + NaCl ($n = 6$). Adjusted p-value < 0.005 indicates treatments with significant variability between means.

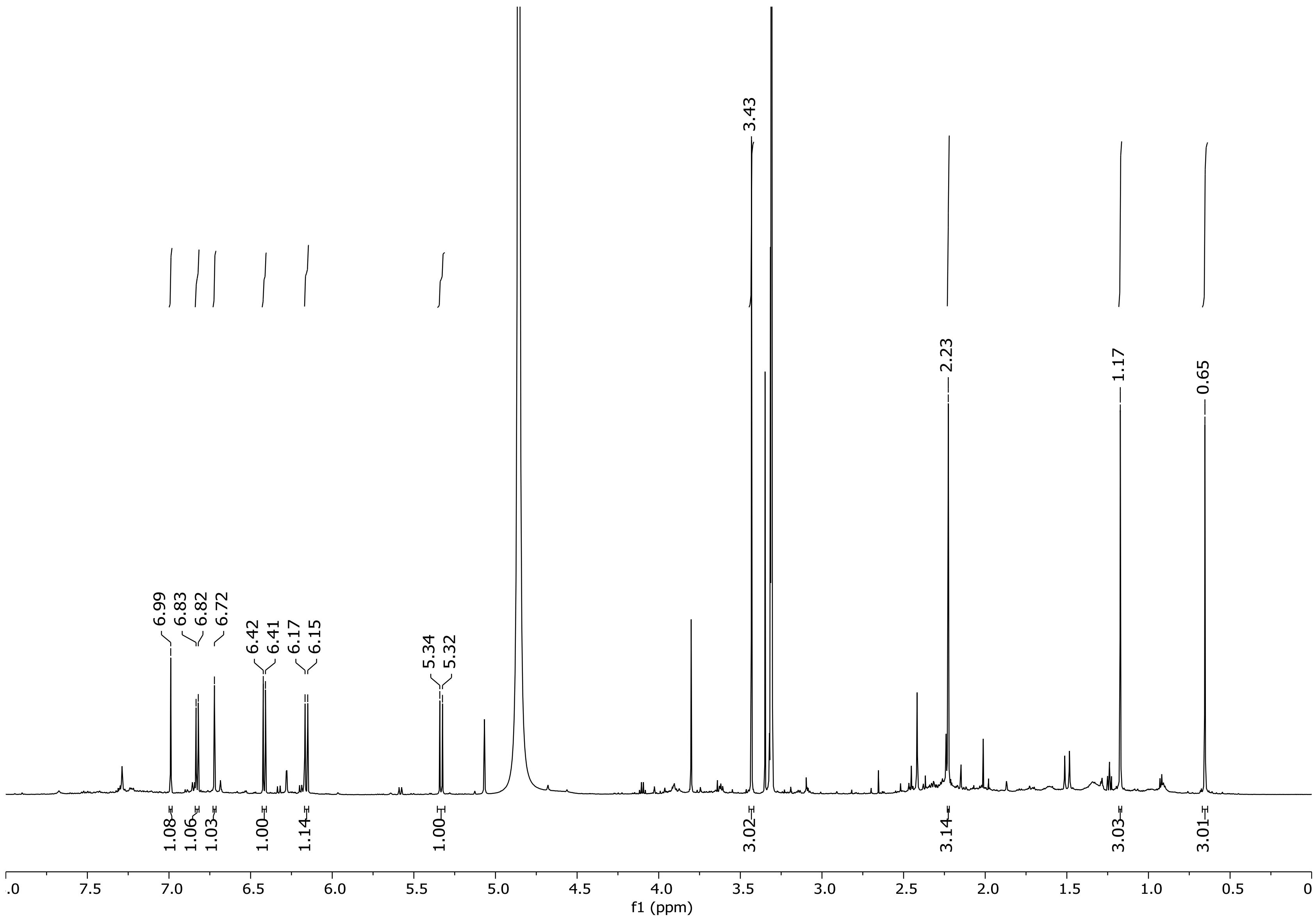

Figure S1. ^{1}H NMR spectrum (600 MHz, CD_3OD) of Primarolide A (**1**).

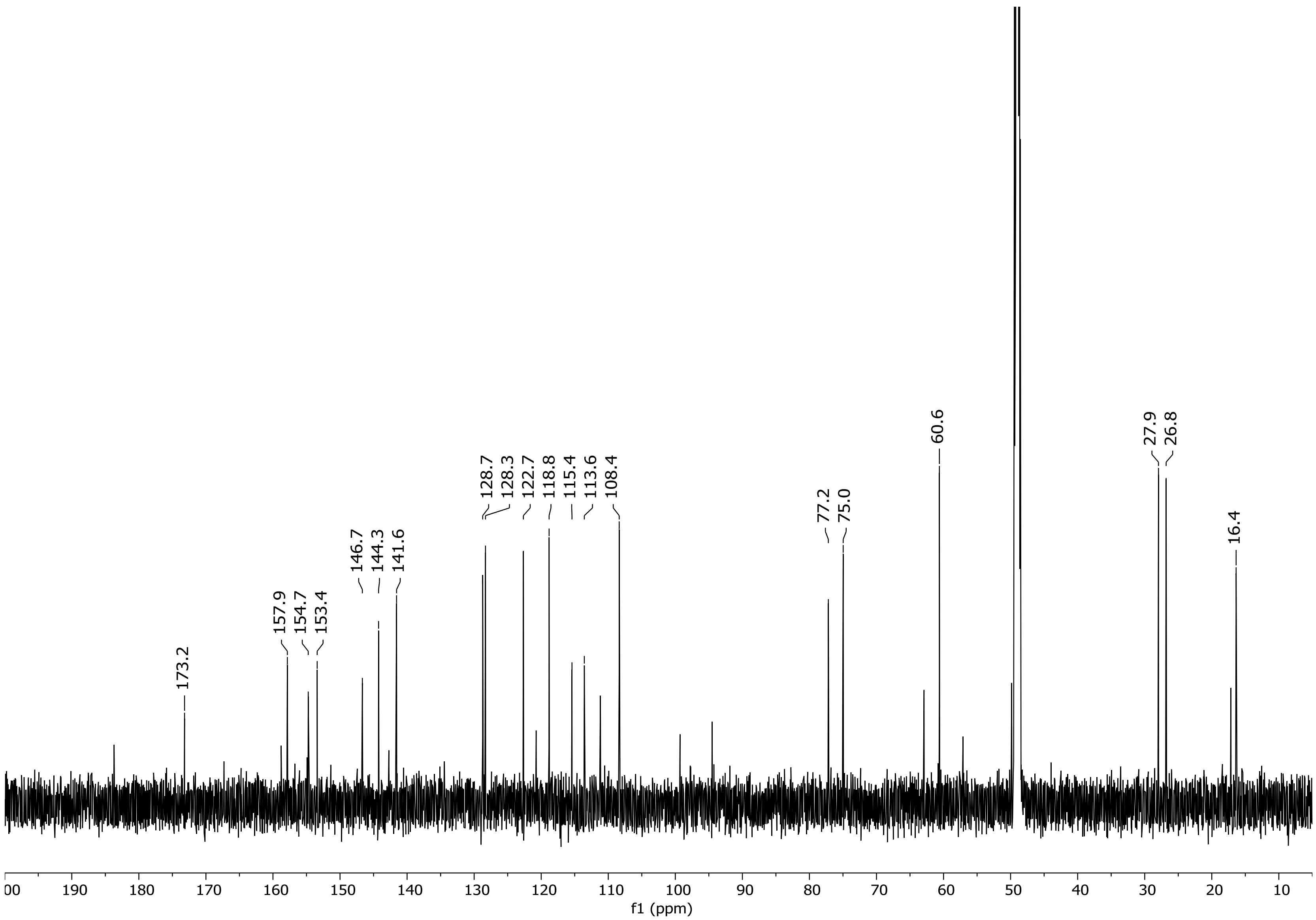

Figure S2. ^{13}C NMR spectrum (150 MHz, CD_3OD) of Primarolide A (**1**).

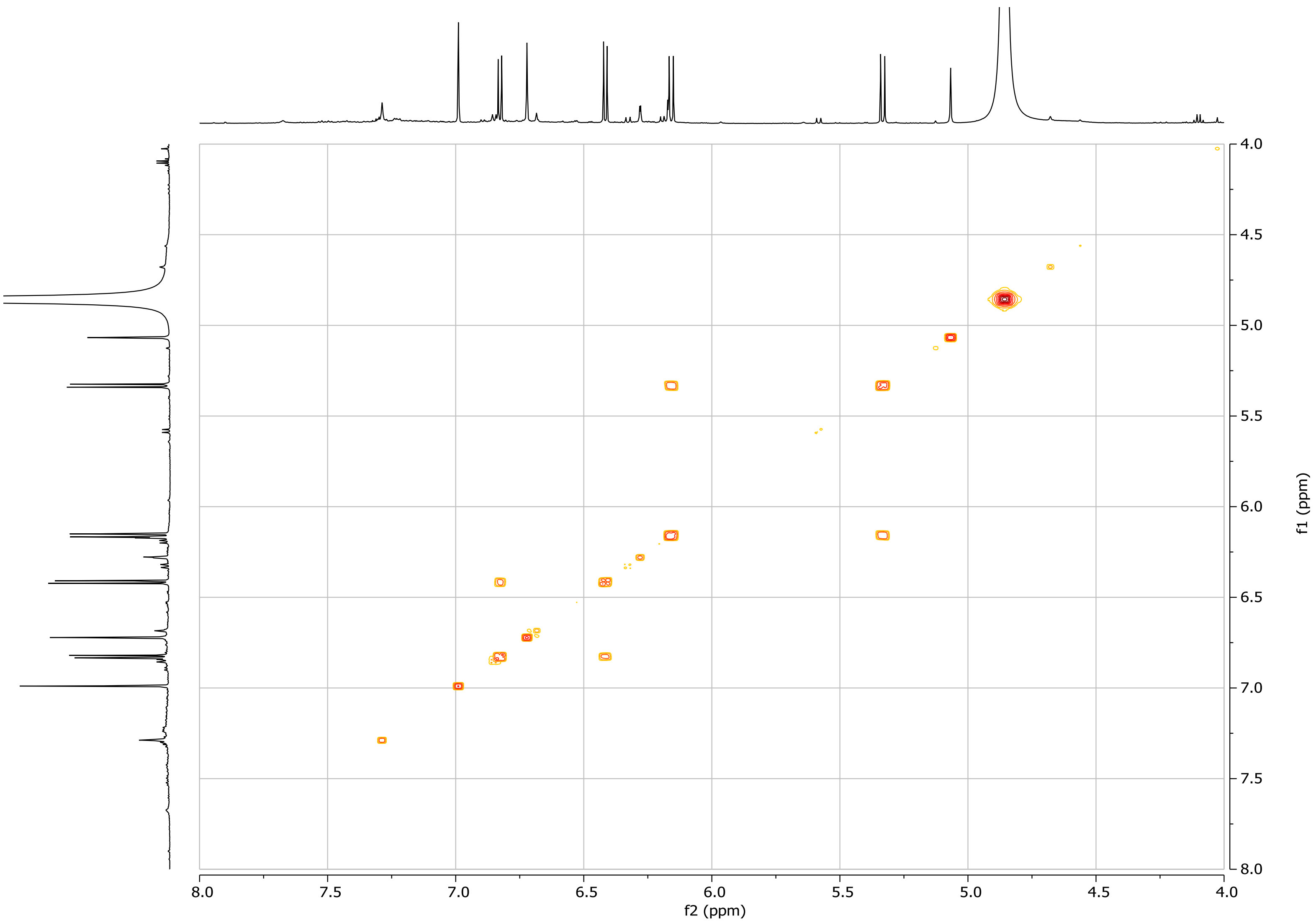

Figure S3. COSY NMR spectrum of Primarolide A (1).

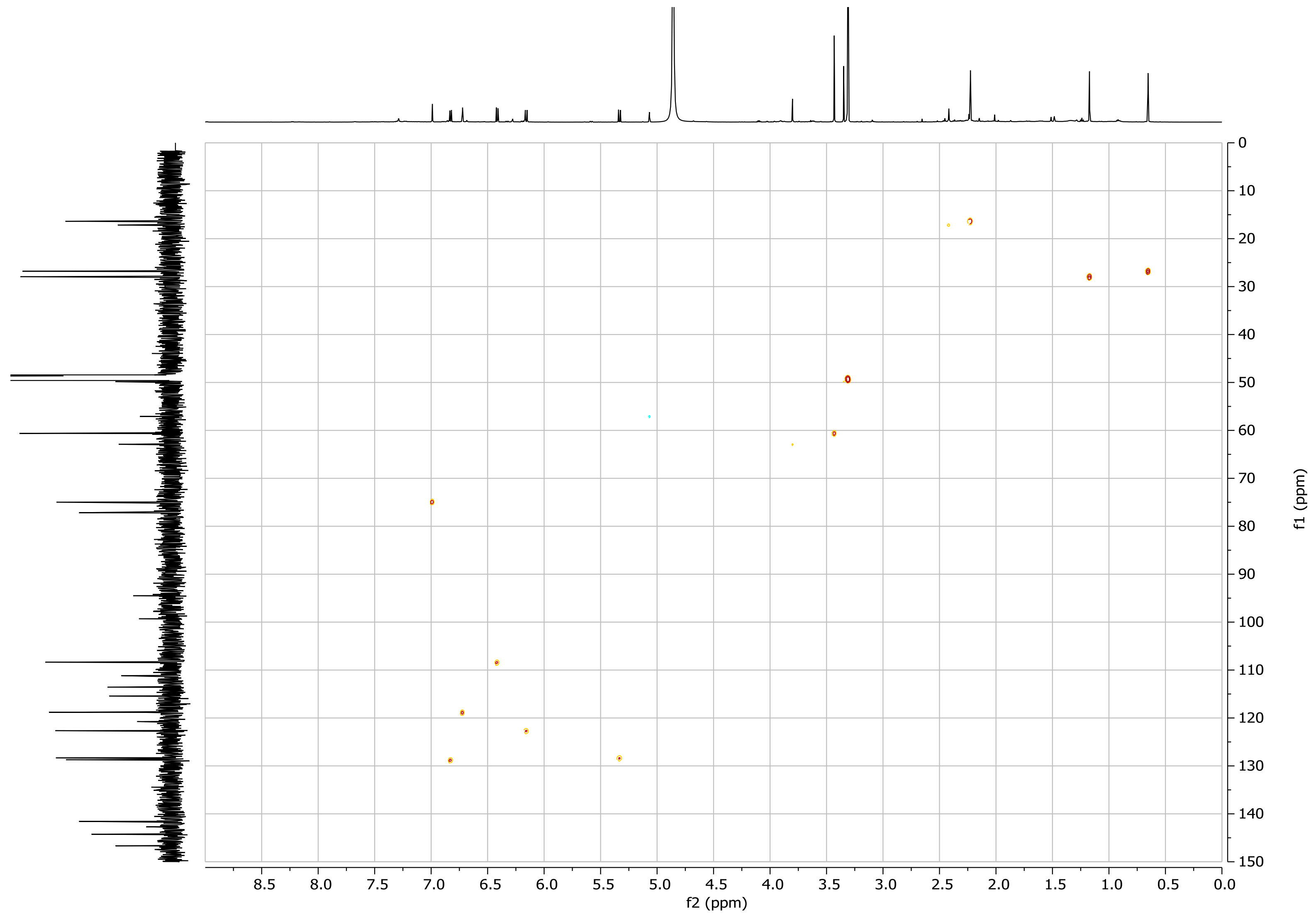

Figure S4. HSQC NMR spectrum of Primarolide A (1).

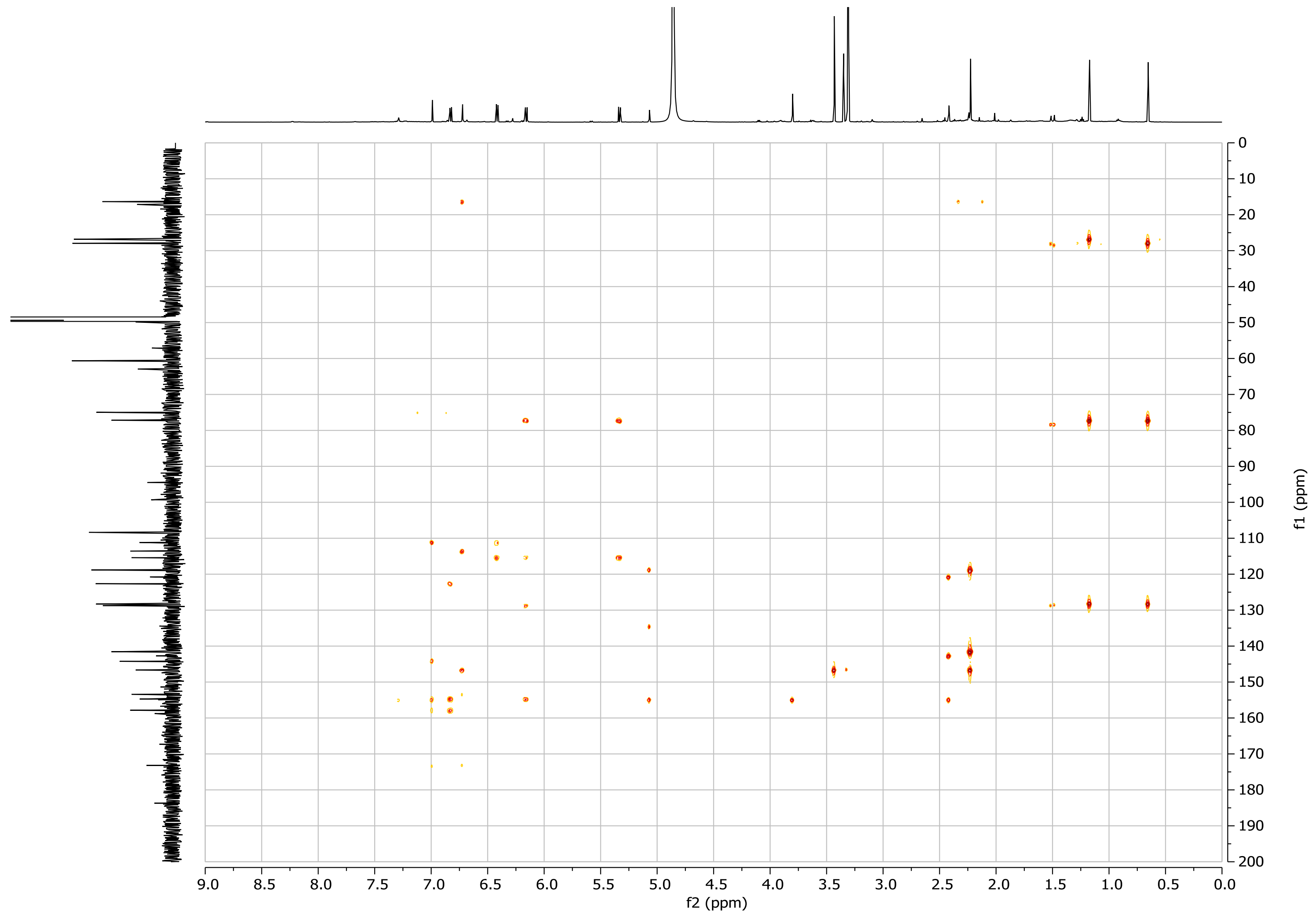

Figure S5. HMBC NMR spectrum of Primarolide A (**1**).

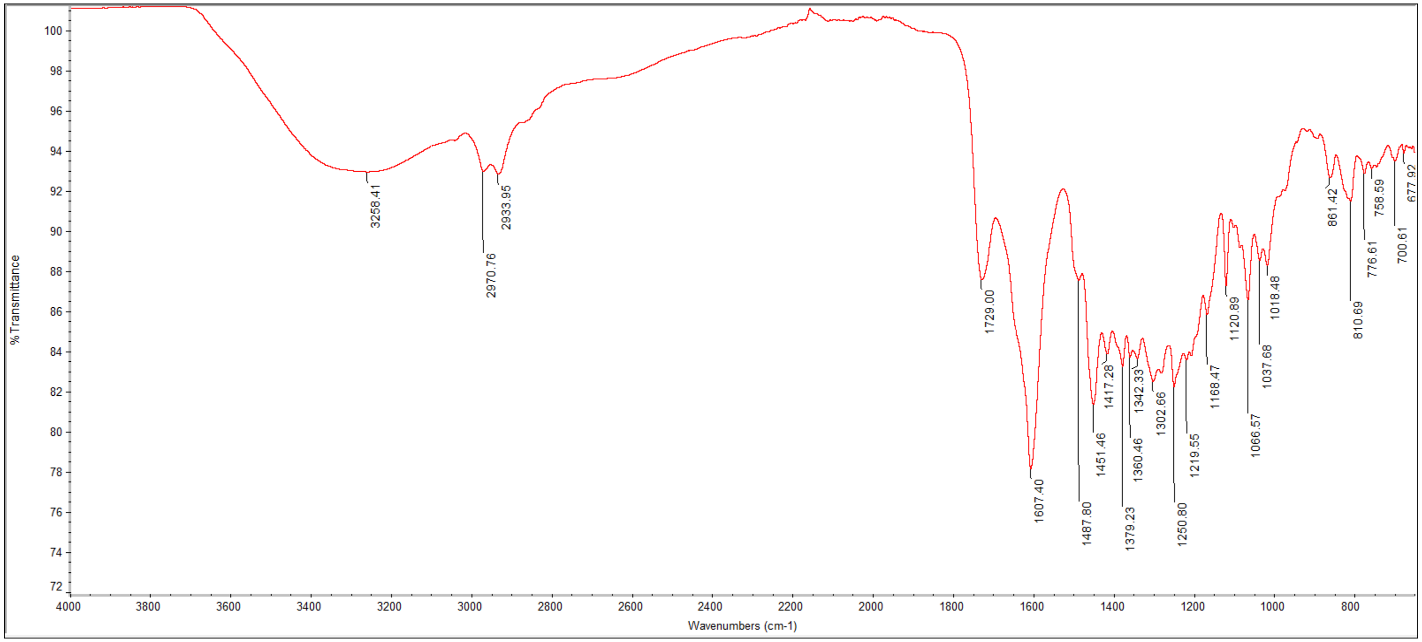

Figure S6. FTIR spectrum of Primarolide A (**1**).

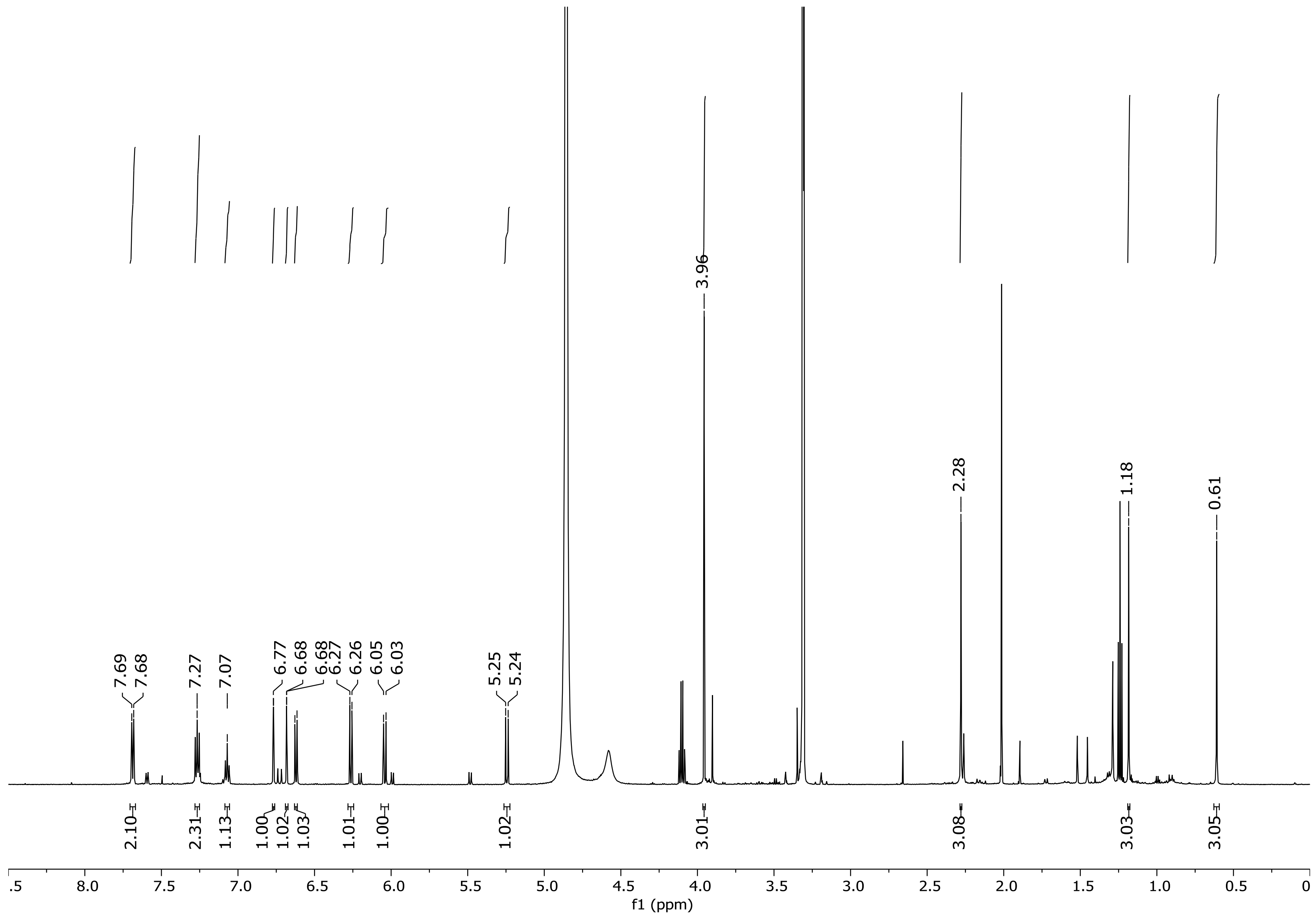

Figure S7. ^{1}H NMR spectrum (600 MHz, CD_3OD) of Primarolide B (**2**).

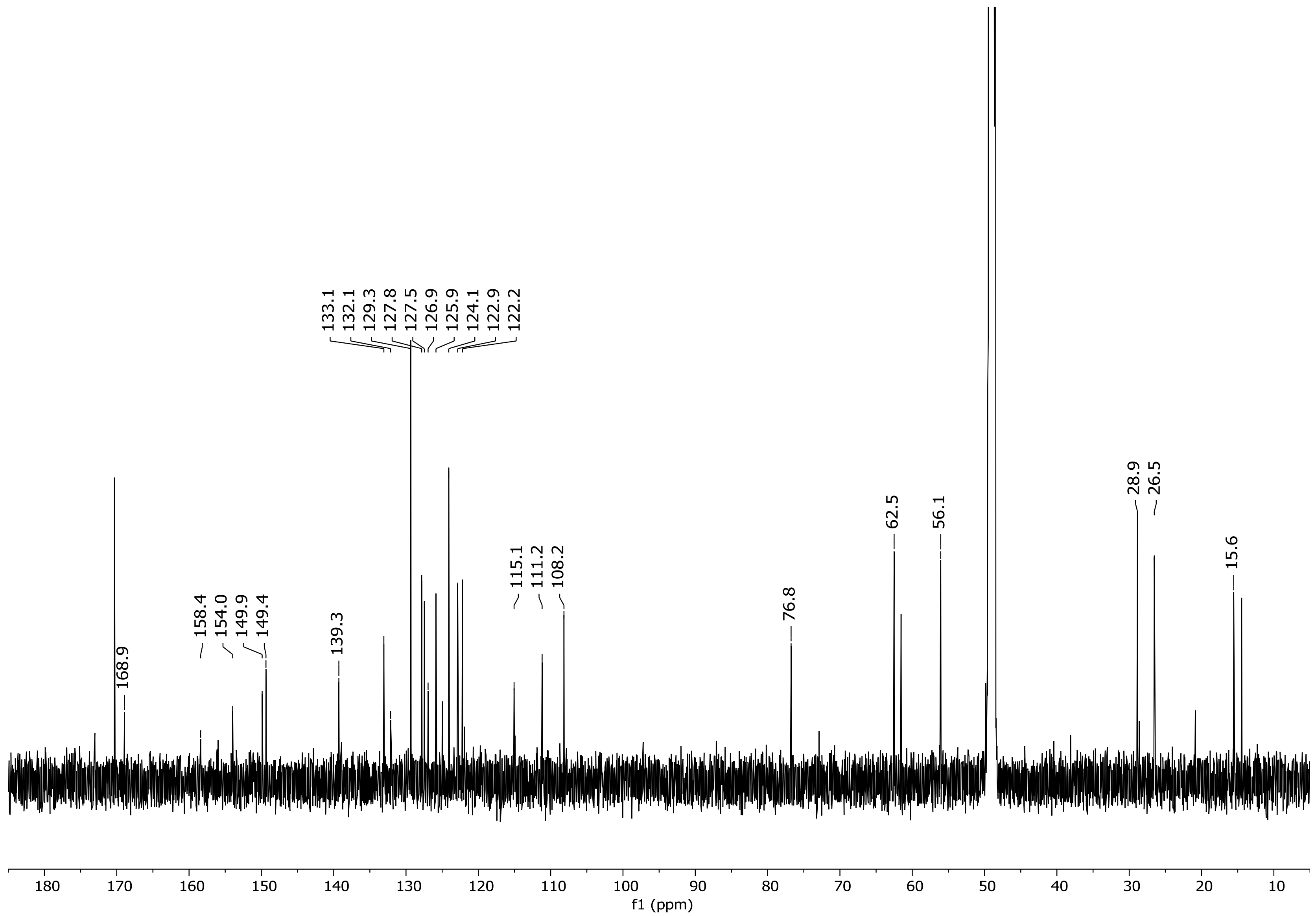

Figure S8. ^{13}C NMR spectrum (150 MHz, CD_3OD) of Primarolide B (**2**).

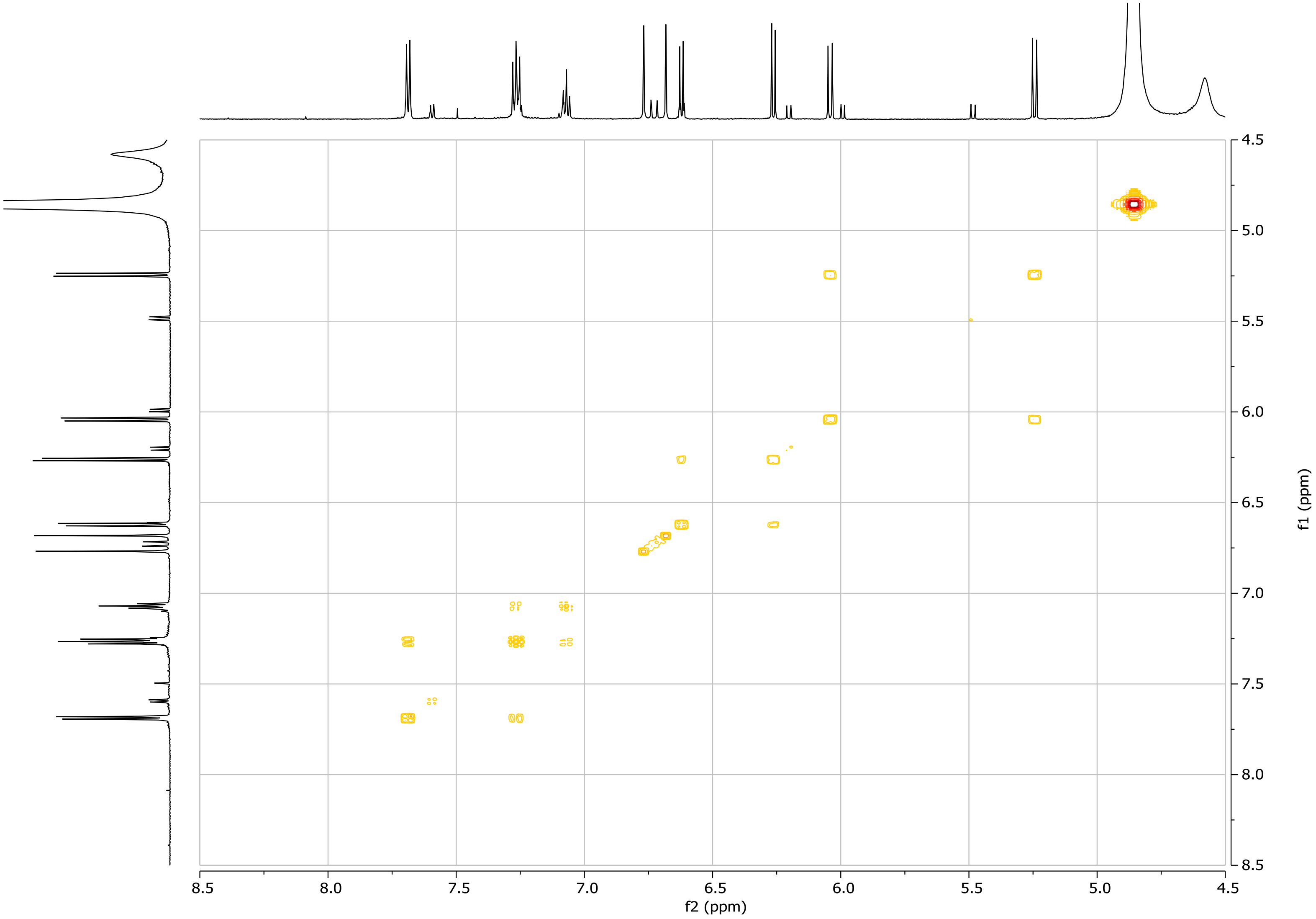

Figure S9. COSY NMR spectrum of Primarolide B (**2**).

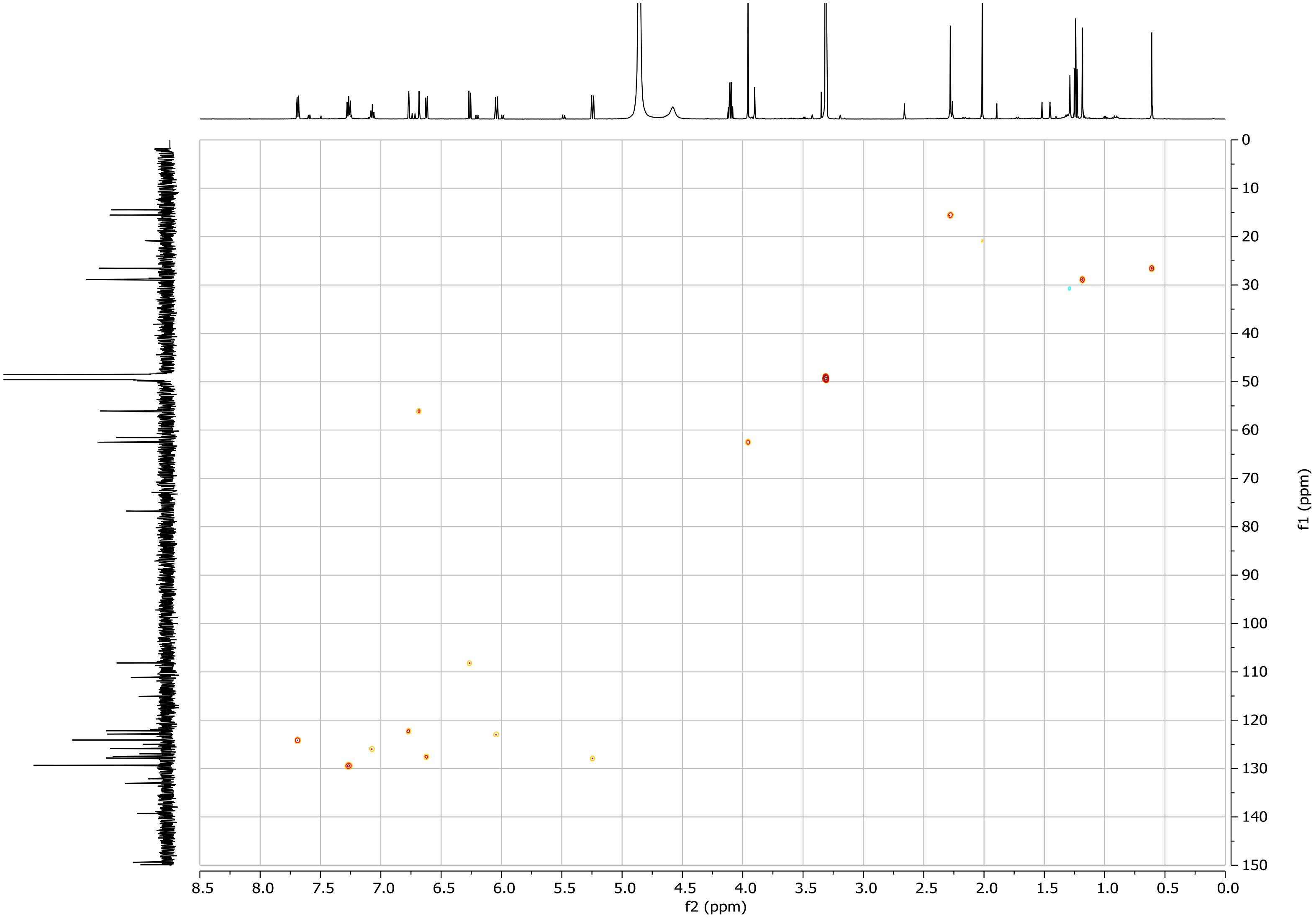

Figure S10. HSQC NMR spectrum of Primarolide B (**2**).

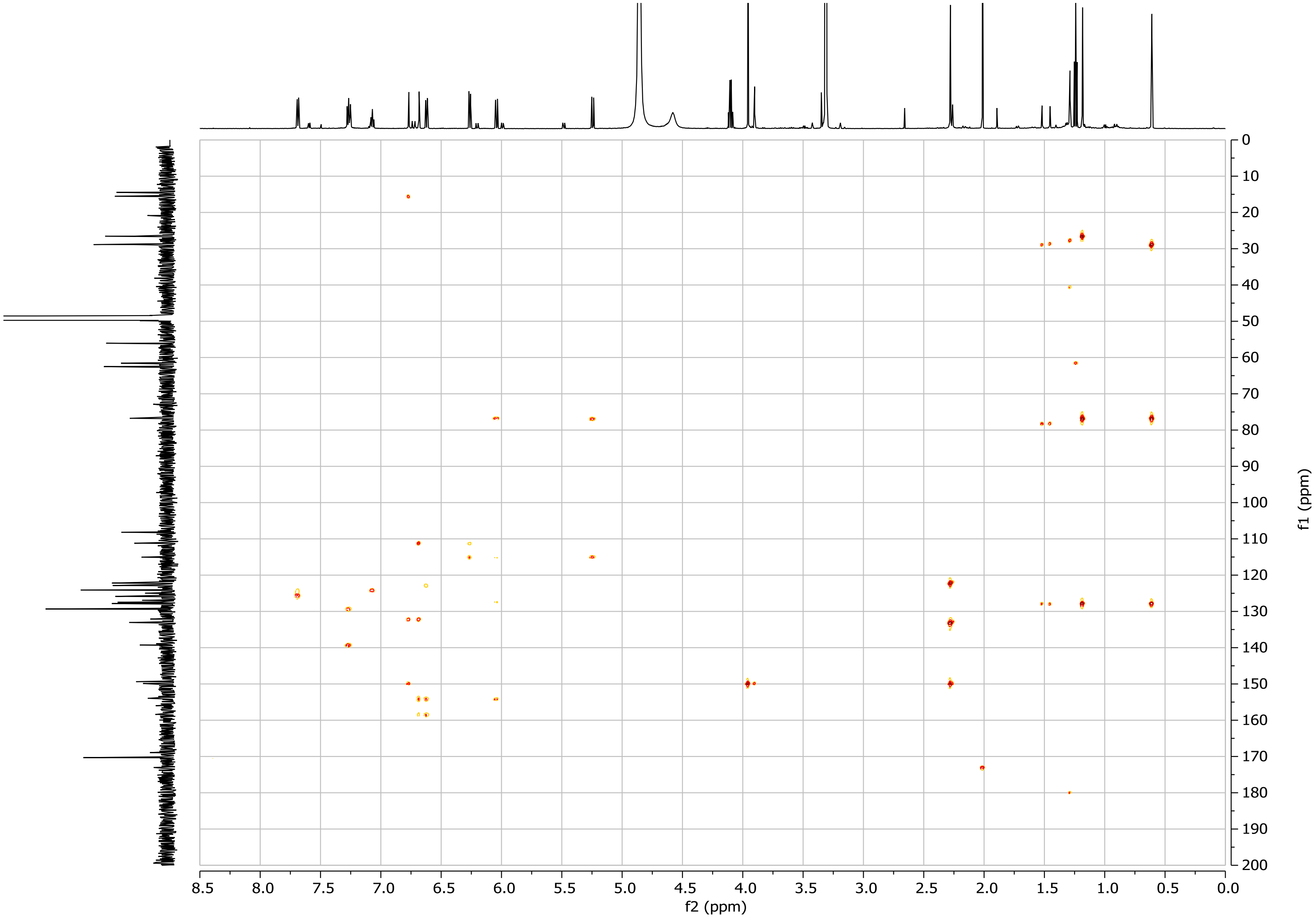

Figure S11. HMBC (8 Hz) NMR spectrum of Primarolide B (**2**).

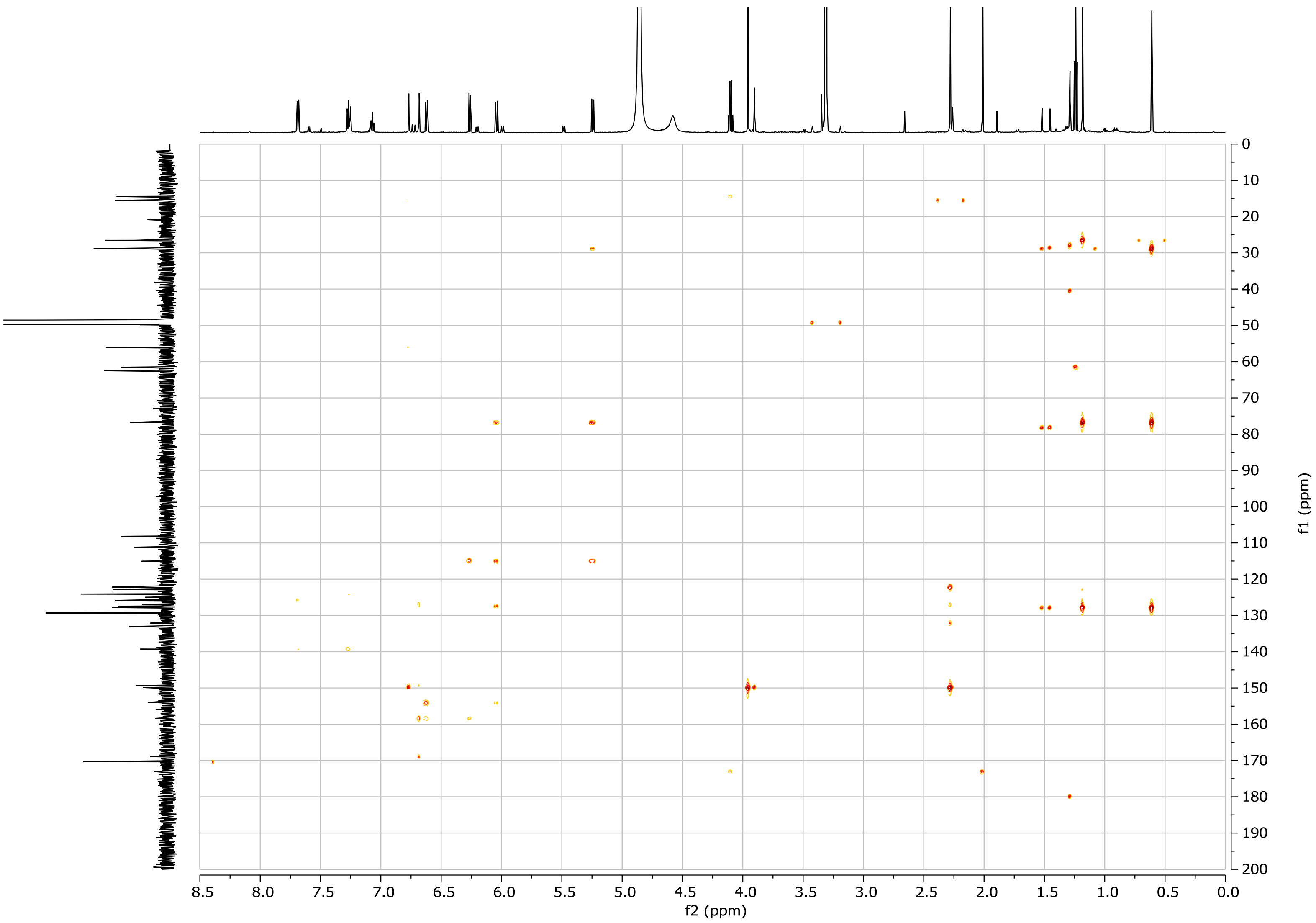

Figure S12. HMBC (3 Hz) NMR spectrum of Primarolide B (**2**).

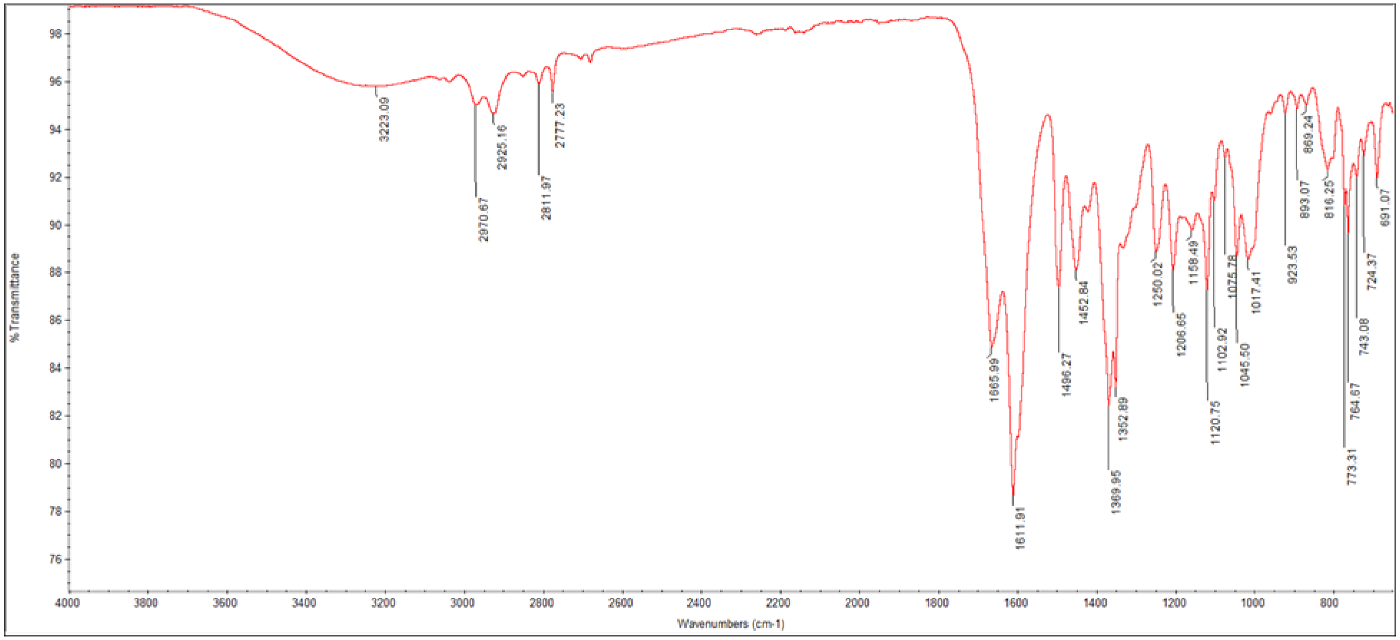

Figure S13. FTIR spectrum of Primarolide B (**2**).
